# Screening for Clinically Significant Nephrolithiasis Based on Simple Health Checkup Clinical and Urine Parameters in General Populations: Multicenter Machine Learning Study

**DOI:** 10.2196/80764

**Published:** 2026-02-19

**Authors:** Hao-Wei Chen, Pei-Siou Wei, Yu-Chen Chen, Jeng-Yih Wu, Chia-I Lin, Yii-Her Chou, Yung-Shun Juan, Wen-Jeng Wu, Chung-Yao Kao, Jung-Ting Lee

**Affiliations:** 1 Department of Urology, Kaohsiung Medical University Chung-Ho Memorial Hospital, Kaohsiung, Taiwan; 2 Graduate Institute of Clinical Medicine, College of Medicine, Kaohsiung Medical University, Kaohsiung, Taiwan; 3 Center for Big Data Research, Kaohsiung Medical University, Kaohsiung, Taiwan; 4 Department of Electrical Engineering, National Sun Yat-sen University, Kaohsiung, Taiwan; 5 United Kaohsiung AI Medical Technology Co, Ltd., Kaohsiung, Taiwan; 6 Regenerative Medicine and Cell Therapy Research Center, Kaohsiung Medical University, Kaohsiung, Taiwan; 7 Department of Internal Medicine, Kaohsiung Medical University Chung-Ho Memorial Hospital, Kaohsiung, Taiwan; 8 Health Management Center, Kaohsiung Medical University Chung-Ho Memorial Hospital, Kaohsiung, Taiwan; 9 College of Medicine, Kaohsiung Medical University, Kaohsiung, Taiwan; 10 School of Medicine, National Sun Yat-sen University, 70 Lien-Hai Road, Kaohsiung, 80424, Taiwan, 886 5252000 ext 4198

**Keywords:** machine learning, nephrolithiasis, health services, software as a medical device, computer-aided diagnosis

## Abstract

**Background:**

Nephrolithiasis affects approximately 15% of the population and often remains undetected in asymptomatic individuals. Current diagnostic approaches rely on imaging tools, such as ultrasound or computed tomography, which are costly, operator dependent, or involve radiation, making them unsuitable for large-scale screening. A standardized, practical, and low-cost screening strategy for early identification of clinically significant kidney stones is still lacking.

**Objective:**

This study aimed to develop a low-cost, rapid screening model for clinically significant nephrolithiasis using machine learning (ML) and simple clinical parameters.

**Methods:**

We conducted a multihospital retrospective study using data from 3 hospitals in Kaohsiung, Taiwan (2012‐2021). Adults without renal colic were included. ML models were trained and tested using 10 routine variables: sex, age, BMI, gout, diabetes, estimated glomerular filtration rate, urine pH, red blood cell count, specific gravity, and bacteriuria. Multiple ML algorithms were trained and evaluated, and the best-performing model was selected based on the area under the receiver operating characteristic curve and the area under the precision-recall curve. To assess model interpretability, Shapley value analysis was performed to determine the relative importance and contribution of each variable to the model’s predictive performance.

**Results:**

Among 6528 participants, the best-performing model achieved an area under the receiver operating characteristic curve of 0.968 (95% CI 0.956‐0.980), an area under the precision-recall curve of 0.936 (95% CI 0.918‐0.953), a sensitivity of 0.873 (95% CI 0.841‐0.904), and a specificity of 0.947 (95% CI 0.935‐0.959). Shapley value analysis identified urine red blood cell count, estimated glomerular filtration rate, and urine specific gravity as the 3 most influential predictors.

**Conclusions:**

This ML-based model enables efficient, noninvasive, and large-scale kidney stone screening using routine health data. It can be integrated into health checkups or telemedicine platforms to facilitate early detection and proactive management. Although the model was developed using an Asian population, future validation in diverse cohorts is warranted to confirm its generalizability.

## Introduction

Approximately 10% of the global population faces a lifetime risk of developing kidney stones [1], and the prevalence of this disease has been steadily rising over the past two decades [2,3]. The most common symptoms include flank pain and hematuria, and kidney and ureteral stones are significant risk factors for chronic kidney disease [4]. A study reported that in the year 2000 alone, the total medical expenditure for treating kidney and ureteral stones in the United States reached US $2.1 billion [5].

Currently, there is no effective tool available for kidney stone screening. Renal ultrasonography is the most commonly used method for detecting kidney stones in clinical settings and emergency departments [5]. While it is a convenient and radiation-free technique, its accuracy is highly dependent on the operator’s skill, thus requiring trained medical professionals for both operation and interpretation. As a result, its use in large-scale screening would demand substantial health care manpower [6,7]. In contrast, abdominal computed tomography (CT) is considered the gold standard for diagnosing kidney and ureteral stones [8]. However, due to its high radiation exposure and limited accessibility for routine or large-scale screening, CT is not suitable for asymptomatic populations or frontline outpatient use [9]. Therefore, existing imaging tools are impractical for large-scale screening of kidney stones. Developing a simple, cost-effective, and accessible screening tool would be highly beneficial for the early detection of kidney stones and for timely medical decision-making.

We previously developed an artificial intelligence (AI) algorithm that uses routine health checkup data, including urine routine and blood creatinine, to preliminarily predict the presence of kidney stones larger than 2 mm in overweight individuals (BMI ≥25 kg/m^2^) [6]. This tool has shown potential for use in kidney stone screening. In this study, we aimed to build upon our prior success by leveraging machine learning (ML) techniques and routine health data to further develop a clinically applicable AI model that can predict clinically significant kidney stones in all patients—not limited to those with BMI >24 kg/m^2^. The models will be validated using patient cohorts from multiple hospitals.

## Methods

### Description of Participants

In this cohort study, clinical data were retrospectively collected from three hospitals located in Kaohsiung, Taiwan: Kaohsiung Medical University Hospital, Kaohsiung Municipal Ta-Tung Hospital, and Kaohsiung Municipal Siaogang Hospital. The data collection period spanned from January 2012 to March 2021.

Patients were categorized into 2 groups based on the presence or absence of nephrolithiasis measuring ≥2 mm. This classification followed the methodology of our previously published ML-based nephrolithiasis screening model developed for overweight and obese individuals [6]. In accordance with prior urological literature, stone fragments smaller than 2 mm are considered clinically insignificant, as they are unlikely to cause stone-related events or require intervention [7]. Therefore, a cutoff of 2 mm was adopted in this study to define clinically significant nephrolithiasis. The presence of stones of 2 mm or larger was confirmed using abdominal CT or a combination of renal ultrasonography and plain abdominal radiography (KUB: kidney, ureter, and bladder), consistent with our prior study protocol [6]. Patients were excluded if they experienced renal colic at the time of data collection, had undergone kidney transplantation, had indwelling foreign bodies in the upper urinary tract (eg, ureteral stents or percutaneous nephrostomy tubes), were receiving renal replacement therapy (hemodialysis or peritoneal dialysis), were aged younger than 18 years, or lacked complete medical records. For model development, data from January 2012 to December 2019 were used for training and validation. An independent test cohort comprising data collected between January 2020 and March 2021 was used for the clinical evaluation of model performance.

### Ethical Considerations

This study was approved by the Institutional Review Board of Kaohsiung Medical University Hospital (KMUHIRB-E(I)-20210331) and conducted in accordance with the principles outlined in the Declaration of Helsinki. The requirement for informed consent was waived due to the retrospective nature of the study and the minimal risk to participants. All data were obtained from existing electronic health records and were fully deidentified before analysis. No personally identifiable information was accessed by the researchers, and data access was restricted to authorized study personnel only. No compensation was provided to participants, as no direct contact or intervention was involved in this retrospective study.

### Variables and Data Processing

A total of 10 predictor variables were included for model development, categorized into sociodemographic, health-related, and clinical domains. The categorical response variable indicated the presence (1) or absence (0) of nephrolithiasis ≥2 mm. Sociodemographic variables included age (numeric) and sex (categorical: male=1 and female =0). Health-related variables comprised BMI (numeric with 2 decimals) and a history of diabetes mellitus and gout (both categorical: with=1 and without=0).

Clinical variables included five parameters derived from urine and blood tests:

1.  Urine specific gravity (SG; numeric with 3 decimals)
2.  Urine pH (numeric with 1 decimal)
3.  Bacteriuria (categorical: present =1, absent =0)
4.  Urine red blood cell (RBC) count (treated as a numeric variable; details described as follows): In Taiwan, urine RBC counts are commonly reported in terms of a range (eg, 6‐10 RBCs per high-power field [HPF]), with the lowest range being “0 to 2 RBCs per HPF.” In this study, the urine RBC data we collected consist of the following ranges: 0 to 2, 3 to 5, 6 to 10, 11 to 25, 26 to 50, 51 to 99, and 100 or greater. As such, it may be treated as a categorical variable in this study. However, to accommodate various range scales used by different hospitals and make the model universally applicable, we adopted the mean of the reported ranges (eg, 8 for the range 6‐10 RBCs per HPF) as the corresponding urine RBC count and treated the variable as numeric (continuous). The exceptions to the rule are the range of “0 to 2” and the ranges whose mean values exceed 100. For the 0 to 2 range, the value 0 is assigned. This is due to an American Urological Association guideline, which defines microhematuria as “3 or more RBCs per HPF on microscopic examination.” Therefore, 0 to 2 RBCs per HPF is generally considered as no hematuria. On the other hand, patients with macroscopic hematuria generally have their urine RBC counts reported as “100 or more RBCs per HPF,” or a range with a mean value exceeding 100, or something equivalent to that effect. As such, for ranges whose mean values exceed 100 RBCs per HPF, we applied the capped value of 200 to maintain consistency across the dataset [10].
5.  Estimated glomerular filtration rate (eGFR), numeric with 1 decimal, calculated using the Isotope Dilution Mass Spectrometry–traceable Modification of Diet in Renal Disease equation: eGFR (mL/min/1.73 m^2^)=175×(Scr)^−1.154^×(age)^−0.203^×(0.742 if female) [8]

For descriptive statistics, the Fisher exact test was used for categorical variables (eg, sex, history of diabetes mellitus, and history of gout), and the Mann-Whitney *U* test was applied for numeric variables (eg, age, BMI, eGFR, urine SG, urine RBC count, and urine pH) between patients with and without nephrolithiasis. To verify the similarity between the training and testing datasets, we compared feature distributions by calculating category percentages for categorical variables and means with SDs for continuous variables. All statistical analyses were performed using the Statistical Package for the Social Sciences for Windows (version 24.0; IBM), and *P* values of less than .05 were considered statistically significant.

### Model Development, Fitting, and Evaluation

ML methods were used to build the models [9]. The logistic regression, the k-nearest neighbors algorithm, the support vector machine, and the random forest (RF) algorithm were used as the standard methods for comparison, while an artificial neural network (ANN) model was developed for deep learning classification. All standard models were implemented in Python using the scikit-learn library (version 1.1.3; scikit-learn developers) with recommended settings. The respective algorithms for each method were run using the recommended parameters [11,12].

For the ANN, implemented in Python, the 10-fold cross-validation protocol was performed on the training set to produce 10 models, each structured as a 9-layer fully connected neural network. These models were averaged into 1 final model. To address the imbalance between stone-positive and stone-negative cohorts, the synthetic minority over-sampling technique, as well as the approach of assigning different weights to the 2 patient groups, was adopted when training our models. In addition, a pronounced sex imbalance among the patient cohorts was observed. To ensure equitable accuracy for the 2 gender groups, 2 separate cutoff thresholds were identified for male and female patients. The 2 thresholds were then standardized to 0.5 by applying gender-specific rescalers to the averaged outputs. The resulting combined structure served as the final ANN model with a unified threshold of 0.5. Additional information is provided in Figure S1 in Multimedia Appendix 1.

Data between January 2012 and December 2019 were used for model training and validation. The data were randomly divided into a training dataset (75%) and a validation dataset (25%). Model training was performed using the training data, while validation data helped identify overfitting or bias. Data registered after January 1, 2020, formed the testing dataset (ie, the holdout dataset) for independent evaluation of model performance. Performance on the testing data was assessed by various metrics, including the area under the receiver operating characteristic (ROC) curve (AUROC), the area under the precision-recall (PR) curve (AUPRC), and net benefit by the decision curve analysis. The optimal thresholds for standard models were based on the Youden index [13] from their respective training ROC curves. For the ANN, the classification threshold was preset at 0.5. These thresholds were used to calculate sensitivity, specificity, positive predictive value, and negative predictive value on the validation and testing sets. Model calibration analysis was also performed to assess how accurately the output probabilities of the models reflect the true likelihood of outcomes.

To examine model stability and fairness across different patient subgroups, additional analyses were performed by individual variables (eg, sex, diabetes, gout, bacteriuria, urine RBC count, age, BMI, and eGFR), where model performance metrics (area under the curve [AUC] and accuracy) were calculated independently for each subgroup.

### Importance of Feature Variables

To evaluate the predictive power of each feature variable, their relative impact on the ANN model output was assessed by applying the Shapley value analysis [14]. Features with notably high impact were selected, and an ANN model was trained using only these variables. The performance of the model was compared with that of the full-variable ANN model to assess whether key features alone provided sufficient predictive power.

## Results

### Characteristics of Study Participants

In the training dataset, 1243 (33.11%) participants had nephrolithiasis, and 2511 (66.89%) participants were without nephrolithiasis of 2 mm or greater, among a total of 3754 (100.0%) participants. The validation dataset had 414 (33.09%) participants with and 837 (66.91%) participants without nephrolithiasis of 2 mm or greater, among a total of 1251 (100.0%) participants. The testing dataset had 400 (26.26%) participants with and 1123 (73.74%) participants without nephrolithiasis of 2 mm or greater, among a total of 1523 (100.0%) participants. The characteristics of the participants in these 3 sets are described in detail in Tables1  2. Statistical similarity among the 3 sets was observed. It is also observed that the mean values (or the “percentages,” in the case of binary variables) of the variables we selected are statistically different between participants with and without nephrolithiasis. The Fisher exact and Mann-Whitney *U* tests were applied to verify the statistical significance of these differences, and the resulting *P* values are also reported in Tables1  2.

**Table 1.** Descriptive analysis of the training, validation, and testing datasets.

	Training set, n (%)				Validation set, n (%)				Testing set, n (%)			
	w/neph^a^ (n=1243, 33.1%)	wo/neph^b^ (n=2511, 66.9 %)	Total (N=3754)	*P* value	w/neph (n=414, 33.1%)	wo/neph (n=837, 66.9%)	Total (N=1251)	*P* value	w/neph (n=400, 26.3%)	wo/neph (n=1123, 73.7%)	Total (N=1523)	*P* value
Gender				.02				.19				<.001
Male	820 (66.0)	1750 (69.7)	2570 (68.5)		273 (65.9)	584 (69.8)	857 (68.5)		250 (62.5)	433 (38.6)	683 (44.8)	
Female	423 (34.0)	761 (30.3)	1184 (31.5)		141 (34.1)	253 (30.2)	394 (31.5)		150 (37.5)	690 (61.4)	840 (55.2)	
Diabetes				<.001				<.001				<.001
With	220 (17.7)	148 (5.9)	368 (9.8)		86 (20.8)	59 (7.1)	145 (11.6)		94 (23.5)	15 (1.3)	109 (7.2)	
Without	1023 (82.3)	2363 (94.1)	3386 (90.2)		328 (79.2)	778 (92.9)	1106 (88.4)		306 (76.5)	1108 (98.7)	1414 (92.8)	
Gout				<.001				<.001				<.001
With	49 (3.9)	16 (0.6)	65 (1.7)		20 (4.8)	3 (0.4)	23 (1.8)		12 (3.0)	4 (0.4)	16 (1.1)	
Without	1194 (96.1%)	2495 (99.4)	3689 (98.3)		394 (95.2)	834 (99.6)	1228 (98.2)		388 (97.0)	1119 (99.6%)	1507 (98.9)	
BAC^c^				<.001				<.001				<.001
With	191 (15.4)	249 (9.9)	440 (11.7)		47 (11.4)	78 (9.3)	125 (10.0)		49 (12.3)	145 (12.9)	194 (12.7)	
Without	1052 (84.6)	2262 (90.1)	3314 (88.3)		367 (88.6)	759 (90.7)	1126 (90.0)		351 (87.7)	978 (87.1)	1329 (87.3)	
Urine RBC count^d^				<.001				<.001				<.001
0-2	219 (17.6)	2265 (90.2)	2484 (66.2)		80 (19.3)	752 (89.8)	832 (66.5)		84 (21.0)	921 (82.0)	1005 (66.0)	
3-5	128 (10.3)	169 (6.7)	297 (7.9)		36 (8.7)	63 (7.52)	99 (7.9)		41 (10.3)	138 (12.3)	179 (11.7)	
6-10	104 (8.4)	44 (1.8)	148 (3.9)		35 (8.5)	13 (1.6)	48 (3.8)		35 (8.8)	30 (2.7)	65 (4.3)	
11-25	153 (12.3)	23 (0.9)	176 (4.7)		56 (13.5)	6 (0.72)	62 (5.0)		43 (10.7)	19 (1.7)	62 (4.1)	
26-50	141 (11.3)	5 (0.2)	146 (3.9)		38 (9.2)	2 (0.24)	40 (3.2)		33 (8.2)	6 (0.5)	39 (2.5)	
51-99	121 (9.7)	1 (0.04)	122 (3.3)		47 (11.4)	0 (0.0)	47 (3.8)		32 (8.0)	3 (0.3)	35 (2.3)	
≥100	377 (30.4)	4 (0.16)	381 (10.1)		122 (29.4)	1 (0.12)	123 (9.8)		132 (33.0)	6 (0.5)	138 (9.1)	

a w/neph: with nephrolithiasis.

b wo/neph: without nephrolithiasis.

c BAC: bacteriuria.

d RBC: red blood cell.

**Table 2.** Characteristics about age, BMI, urine pH, estimated glomerular filtration rate (eGFR), and urine red blood cell (RBC) count of patients in the training, validation, and testing datasets.

	Training set, mean (SD) [95% CI]			Validation set, mean (SD) [95% CI]			Testing set, mean (SD) [95% CI]		
	w/neph^a^	wo/neph^b^	*P* value	w/neph	wo/neph	*P* value	w/neph	wo/neph	*P* value
Age	55.92 (13.30) [55.15‐56.68]	50.92 (10.10) [50.51‐51.33]	<.001	55.53 (13.47) [54.34‐56.71]	51.36 (10.07) [50.73‐51.98]	<.001	57.31 (13.80) [55.95‐58.66]	45.52 (9.32) [44.97‐46.07]	<.001
eGFR	74.67 (29.23) [72.98‐76.35]	86.31 (17.83) [85.58‐87.03]	<.001	73.38 (30.44) [70.70‐76.06]	85.95 (17.64) [84.86‐87.04]	<.001	69.47 (26.74) [66.84‐72.10]	95.03 (19.18) [93.90‐96.15]	<.001
BMI	25.77 (3.81) [25.55‐25.99]	24.64 (3.63) [24.50‐24.79]	<.001	25.91 (4.02) [25.56‐26.27]	24.67 (3.59) [24.45‐24.89]	<.001	26.65 (4.82) [26.18‐27.13]	23.68 (3.86) [23.45‐23.90]	<.001
Urine pH	6.03 (0.79) [5.98‐6.07]	6.03 (0.74) [6.00‐6.06]	.93	6.07 (0.80) [5.99‐6.15]	6.01 (0.75) [5.96‐6.06]	.21	6.11 (0.84) [6.02‐6.19]	6.02 (0.79) [5.97‐6.06]	.06
Urine RBC count	75.42 (84.64) [70.55‐80.30]	1.03 (8.83) [0.67‐1.39]	<.001	74.93 (84.60) [67.48‐82.38]	0.83 (6.82) [0.41‐1.26]	<.001	78.18 (87.85) [69.54‐86.82]	2.48 (15.48) [1.58‐3.39]	<.001
Urine SG^c^	1.014 (0.006) [1.0137‐1.0144]	1.018 (0.007) [1.018‐1.0185]	<.001	1.014 (0.007) [1.013‐1.0145]	1.018 (0.007) [1.018‐1.0185]	<.001	1.015 (0.008) [1.014‐1.0154]	1.018 (0.006) [1.018‐1.0187]	<.001

a w/neph: with nephrolithiasis.

b wo/neph: without nephrolithiasis.

c SG: specific gravity.

### Model Performance

The ANN model we developed demonstrated strong discriminatory power in identifying participants with kidney or ureteral stones, using only limited information typically collected at an initial clinical visit. It achieved validation and testing AUROCs of 0.970 and 0.968 and validation and testing AUPRCs of 0.960 and 0.936, respectively. Using a cutoff value of 0.5, the model showed high sensitivity, specificity, positive predictive value, and negative predictive value in both validation and testing sets, with overall correction rates of 92.3% and 92.7%, respectively. These performance measures indicate that the model achieved a good balance in identifying both positive and negative cases and is able to render both high precision and high recall.

The performance of models obtained using standard methods appeared to be somewhat inferior to that of the ANN model, with the exception of the RF algorithm, which performed equally well. The logistic regression, k-nearest neighbors, and support vector machine (linear kernel) models had testing AUROCs of 0.933, 0.916, and 0.910, respectively, and testing AUPRCs of 0.822, 0.876, and 0.790, respectively. The RF model achieved AUROC and AUPRC of 0.965 and 0.934, which are almost the same as those of the ANN model. The overall correction rate was 90.5%. Despite the high AUROC and AUPRC scores, the RF model showed unbalanced performance between sensitivity and specificity, a pattern also observed in the other conventional models.

**Figure 1.**
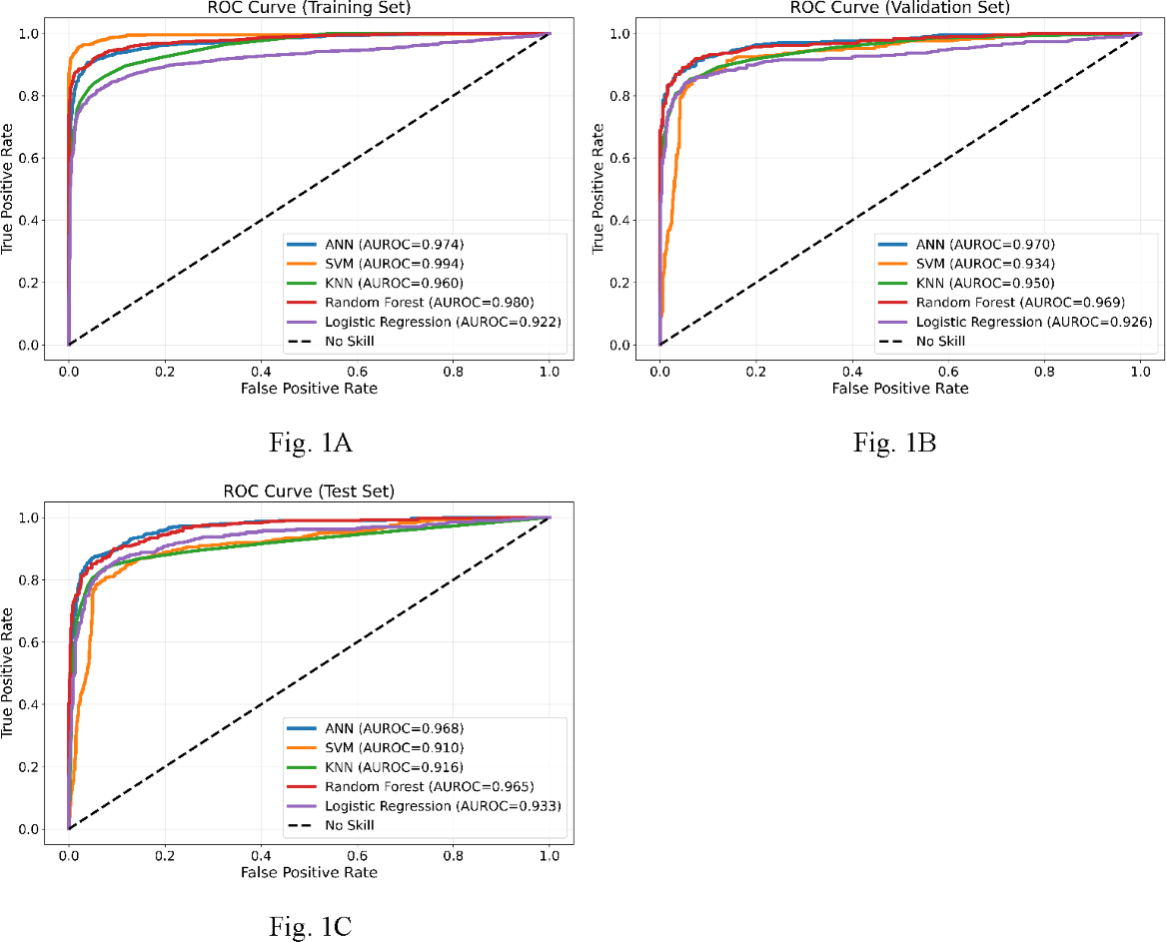
The receiver operating characteristic (ROC) curves of the artificial neural network (ANN), random forest, k-nearest neighbor (KNN), support vector machine (SVM), and logistic regression models. (A) Training ROC curves. (B) Validation ROC curves. (C) Testing ROC curves. AUROC: area under the receiver operating characteristic curve.

**Figure 2.**
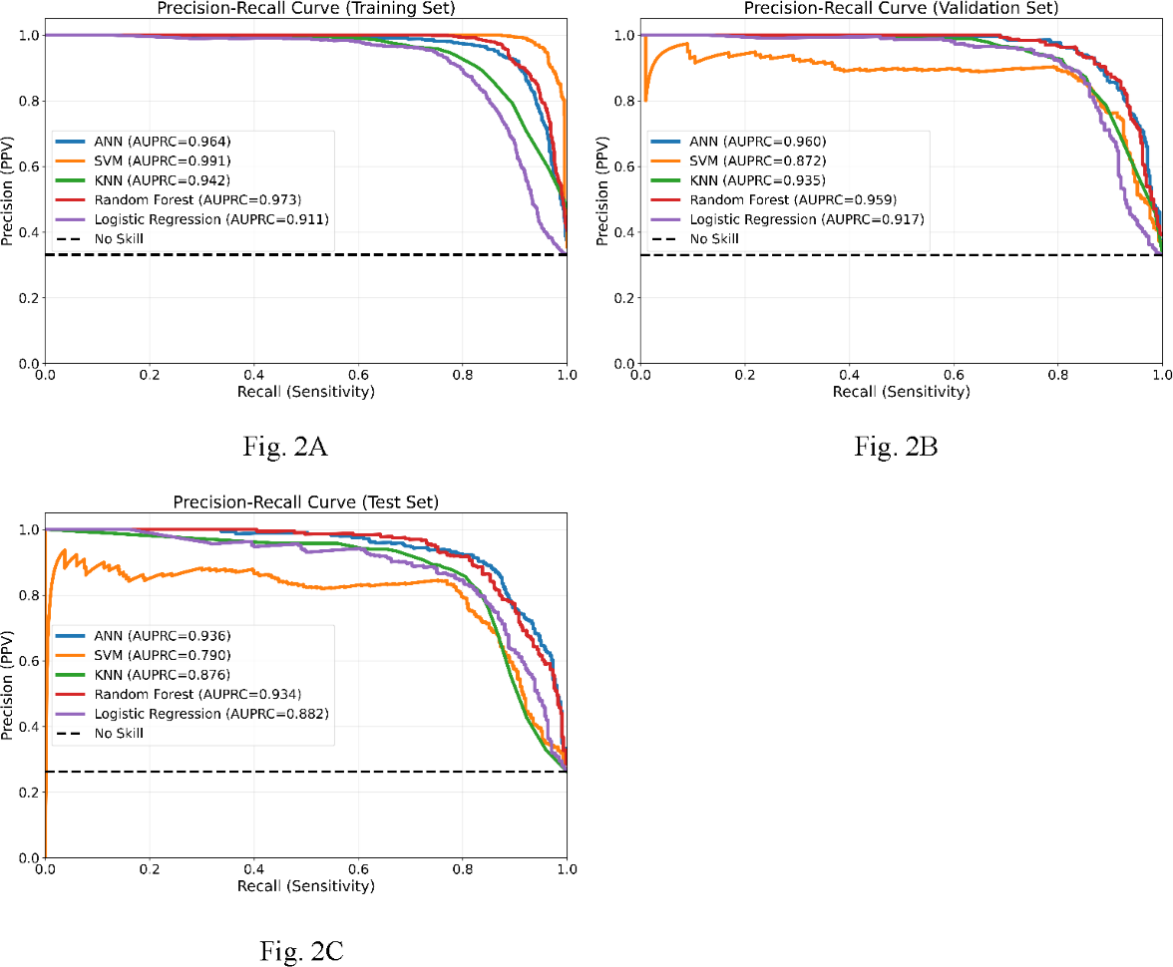
Precision-recall (PR) curves of the artificial neural network (ANN), random forest, k-nearest neighbor (KNN), support vector machine (SVM), and logistic regression models. (A) Training PR curves. (B) Validation PR curves. (C) Testing PR curves. AUPRC: area under the precision-recall curve.

Table 3 summarizes the performance data of these 5 models. Figure 1A-C presents their training, validation, and testing ROC curves, while Figure 2A-C presents the respective PR curves.

**Table 3.** Summary of model performances.

Model and dataset	AUROC^a^ (95% CI)	AUPRC^b^ (95% CI)	Accuracy (95% CI)	Sensitivity (95% CI)	Specificity (95% CI)	PPV^c^ (95% CI)	NPV^d^ (95% CI)
ANN^e^							
Training	0.974 (0.968‐0.980)	0.964 (0.956‐0.971)	0.943 (0.935‐0.951)	0.907 (0.890‐0.923)	0.961 (0.954‐0.968)	0.920 (0.903‐0.937)	0.954 (0.946‐0.963)
Validation	0.970 (0.958‐0.982)	0.960 (0.946‐0.973)	0.923 (0.908‐0.937)	0.889 (0.860‐0.918)	0.939 (0.923‐0.956)	0.878 (0.846‐0.911)	0.945 (0.928‐0.962)
Testing	0.968 (0.956‐0.98)	0.936 (0.918‐0.953)	0.927 (0.915‐0.939)	0.873 (0.841‐0.904)	0.947 (0.935‐0.959)	0.853 (0.819‐0.888)	0.954 (0.941‐0.968)
LR^f^							
Training	0.922 (0.911‐0.934)	0.911 (0.899‐0.922)	0.900 (0.891‐0.910)	0.817 (0.796‐0.839)	0.942 (0.932‐0.951)	0.874 (0.855‐0.893)	0.912 (0.902‐0.923)
Validation	0.926 (0.906‐0.945)	0.917 (0.897‐0.936)	0.908 (0.892‐0.924)	0.843 (0.808‐0.878)	0.940 (0.924‐0.956)	0.875 (0.842‐0.907)	0.924 (0.906‐0.942)
Testing	0.933 (0.916‐0.949)	0.882 (0.859‐0.904)	0.909 (0.895‐0.924)	0.800 (0.761‐0.839)	0.948 (0.935‐0.961)	0.847 (0.810‐0.883)	0.930 (0.915‐0.945)
KNN^g^							
Training	0.960 (0.954‐0.966)	0.942 (0.932‐0.951)	0.905 (0.896‐0.914)	0.861 (0.842‐0.880)	0.927 (0.917‐0.937)	0.853 (0.834‐0.873)	0.931 (0.921‐0.941)
Validation	0.950 (0.936‐0.963)	0.935 (0.918‐0.952)	0.899 (0.882‐0.915)	0.862 (0.829‐0.896)	0.916 (0.898‐0.935)	0.836 (0.801‐0.871)	0.931 (0.914‐0.948)
Testing	0.916 (0.896‐0.936)	0.876 (0.853‐0.899)	0.892 (0.876‐0.907)	0.848 (0.812‐0.883)	0.907 (0.890‐0.924)	0.765 (0.726‐0.805)	0.944 (0.930‐0.957)
SVM^h^							
Training	0.994 (0.991‐0.996)	0.991 (0.987‐0.994)	0.972 (0.966‐0.977)	0.957 (0.946‐0.969)	0.979 (0.973‐0.984)	0.957 (0.945‐0.968)	0.979 (0.973‐0.985)
Validation	0.934 (0.917‐0.949)	0.872 (0.848‐0.895)	0.828 (0.807‐0.849)	0.930 (0.905‐0.955)	0.778 (0.750‐0.806)	0.674 (0.636‐0.713)	0.957 (0.942‐0.973)
Testing	0.910 (0.891‐0.929)	0.790 (0.761‐0.818)	0.722 (0.700‐0.745)	0.920 (0.893‐0.947)	0.652 (0.624‐0.680)	0.485 (0.449‐0.520)	0.958 (0.944‐0.972)
RF^i^							
Training	0.980 (0.975‐0.984)	0.973 (0.966‐0.979)	0.939 (0.931‐0.946)	0.919 (0.904‐0.934)	0.949 (0.940‐0.957)	0.899 (0.882‐0.915)	0.959 (0.952‐0.967)
Validation	0.969 (0.958‐0.979)	0.959 (0.945‐0.972)	0.924 (0.909‐0.939)	0.918 (0.891‐0.944)	0.927 (0.910‐0.945)	0.862 (0.830‐0.894)	0.958 (0.944‐0.972)
Testing	0.965 (0.954‐0.975)	0.934 (0.916‐0.951)	0.905 (0.890‐0.920)	0.900 (0.871‐0.929)	0.907 (0.890‐0.924)	0.774 (0.736‐0.812)	0.962 (0.951‐0.974)

a AUROC: area under the receiver operating characteristic curve.

b AUPRC: area under the precision-recall curve.

c PPV: positive predictive value.

d NPV: negative predictive value.

e ANN: artificial neural network.

f LR: logistic regression.

g KNN: k-nearest neighbor.

h SVM: support vector machine.

i RF: random forest.

**Figure 3.**
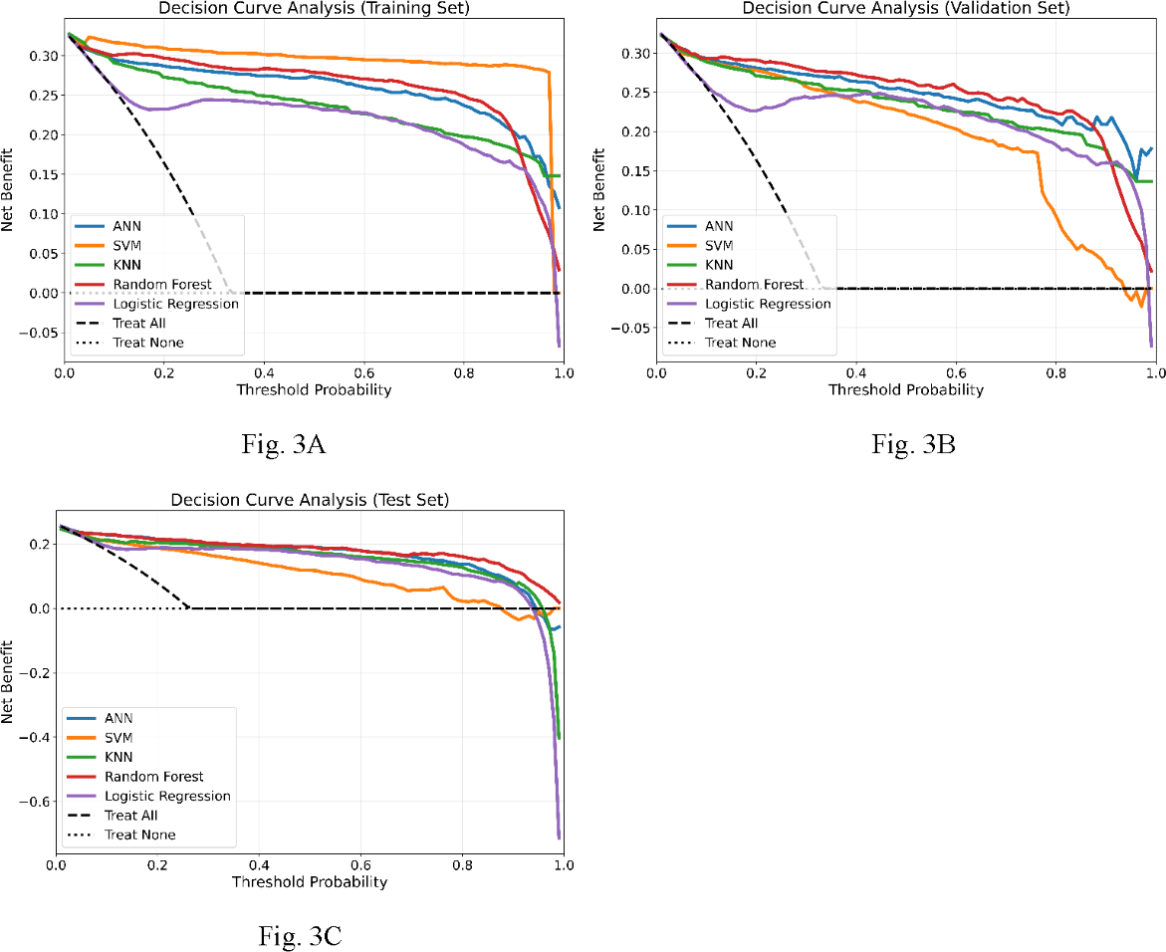
Decision curve analysis for the artificial neural network (ANN), random forest, k-nearest neighbor (KNN), support vector machine (SVM), and logistic regression models. (A) Net benefits over the training set. (B) Net benefits over the validation set. (C) Net benefits over the testing set.

**Figure 4.**
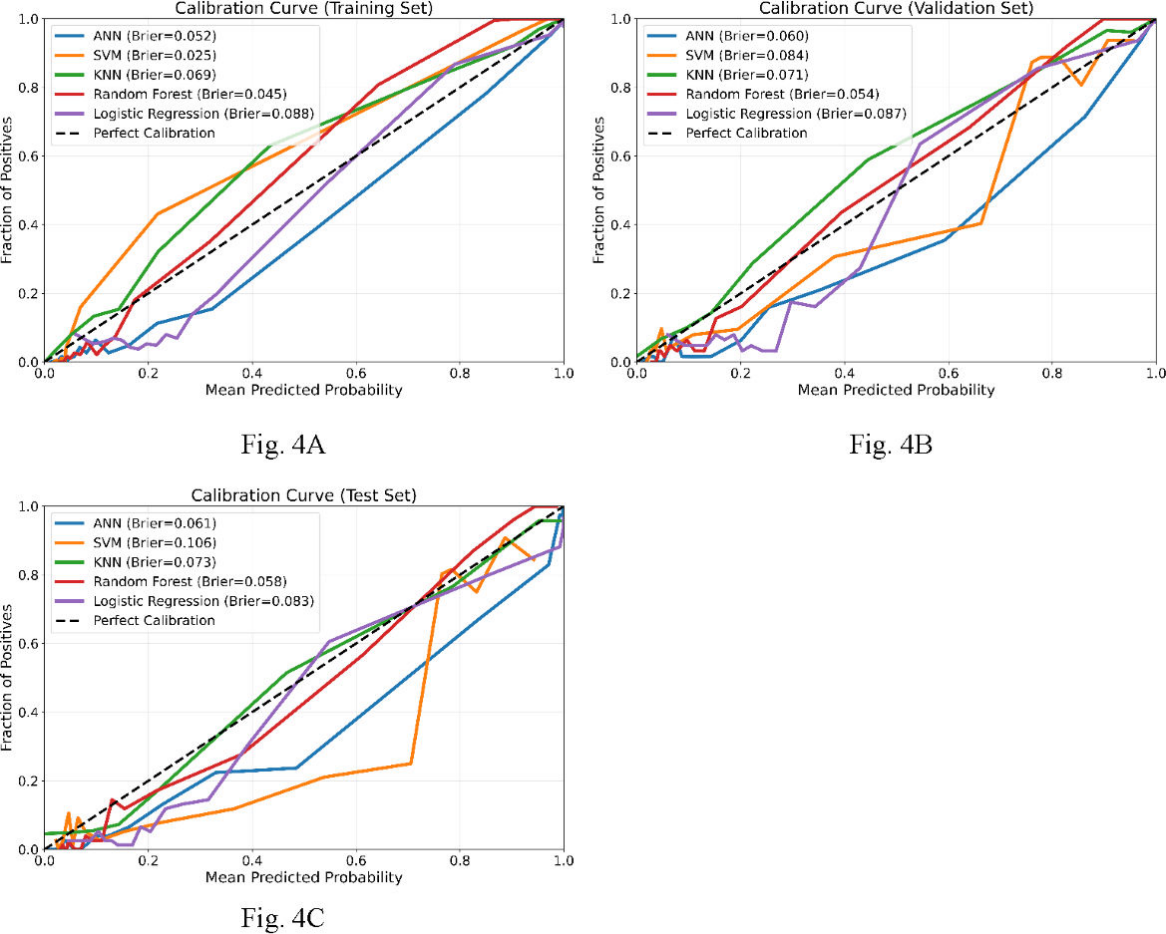
Model calibration analysis for the artificial neural network (ANN), random forest, k-nearest neighbor (KNN), support vector machine (SVM), and logistic regression (LR) models. (A) Over the training set. (B) Over the validation set. (C) Over the testing set.

Regarding the practical relevance of models’ predicted probabilities, the results of decision curve analysis and model calibration analysis are shown in Figure 3A-C and Figure 4A-C, respectively. Figure 3 indicates that all models rendered higher net benefits than those of the baseline strategies (“treat all” and “treat none”) over a large range of threshold probabilities. Among them, the performance of the ANN and RF models appeared to be superior to the others over the validation and testing datasets. Figure 4 indicates that the predicted probabilities of all models were somewhat deviated from the actual events. As such, despite exhibiting good performance for identifying nephrolithiasis, further calibration of the model outputs appears to be required. To further evaluate the robustness and generalizability of the ANN model, subgroup analyses were conducted based on demographic and clinical characteristics.

In the subgroup analysis, the ANN model achieved an AUC of 0.965 and an accuracy of 0.917 in male patients and an AUC of 0.970 and an accuracy of 0.936 in female patients. Similar consistent performances were observed across other subgroups. In patients with diabetes, the model achieved an AUC of 0.961 and an accuracy of 0.890, whereas in those without diabetes, the AUC and accuracy were 0.963 and 0.930, respectively. In patients with gout, the model reached an AUC of 1.000 and accuracy of 1.000, while in those without gout, the AUC was 0.967, and the accuracy was 0.926. For patients with bacteriuria, the AUC was 0.967, and the accuracy was 0.926, compared with 0.968 and 0.929 in those without bacteriuria. Among urine RBC subgroups, the AUC and accuracy were 0.915 and 0.942 for 0 to 2 RBCs, 0.936 and 0.866 for 3 to 5 RBCs, 0.950 and 0.908 for 6 to 10 RBCs, and 0.936 and 0.916 for 10 or more RBCs. By age category, the AUC and accuracy were 0.972 and 0.949 for patients aged 18 to 40 years, 0.956 and 0.923 for those aged 40 to 65 years, and 0.916 and 0.907 for those older than 65 years. For BMI subgroups, the model achieved 0.966 and 0.934 for BMI less than 25 kg/m^2^, 0.961 and 0.918 for BMI of 25 to 30 kg/m^2^, and 0.964 and 0.913 for BMI of 30 kg/m^2^ or greater. Finally, among patients with eGFR less than 60, the AUC and accuracy were 0.992 and 0.981, compared with 0.953 and 0.921 for those with eGFR greater than 60.

### Importance of Feature Variables

**Figure 5.**
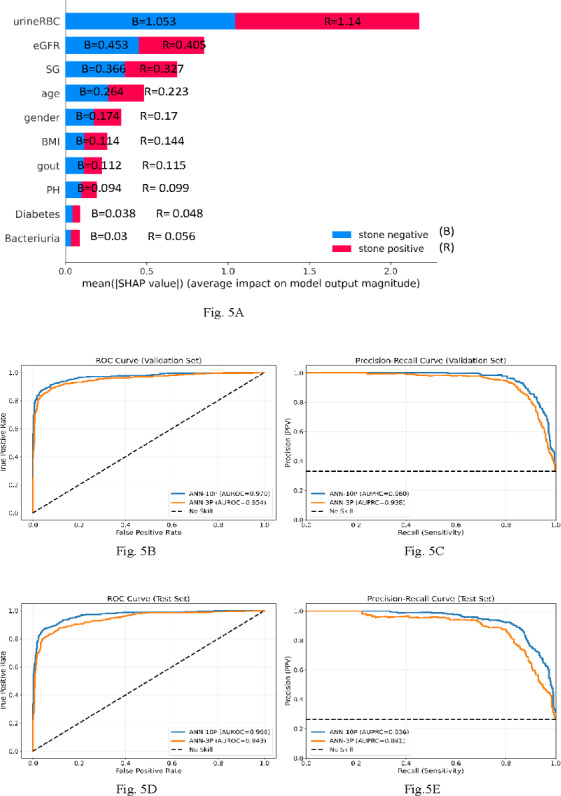
Results of the Shapley value analysis and receiver operating characteristic (ROC), precision-recall curves of the artificial neural network (ANN) models. (A) Summary plot of the Shapley values. (B) Validation ROC curves. (C) Validation PR curves. (D) Testing ROC curves. (E) Testing PR curves. AUROC: area under the receiver operating characteristic curve; AUPRC: area under the precision-recall curve; eGFR: estimated glomerular filtration rate; SG: specific gravity; SHAP: Shapley Additive Explanations; urineRBC: urine red blood cell.

The results of the Shapley value analysis for the ANN model are shown in Figure 5A, which indicates that the predictive power of the model was predominantly driven by three features: urine RBC count, eGFR, and urine SG. An ANN model trained using only these 3 predictors (referred to as “ANN-3P” thereafter, as opposed to “ANN-10P” for the original 10-predictor ANN model) achieved validation and testing AUROCs of 0.954 and 0.943, and validation and testing AUPRCs of 0.938 and 0.891, respectively; the performance is competitive with that of the ANN-10P model. Figure 5B-E displays the ROC and PR curves of the 2 models, and a comprehensive performance comparison is summarized in Table 4. The outstanding performance of the ANN-3P model confirms the predictive power rendered by the 3 key feature variables.

**Table 4.** Performance comparison between artificial neural network (ANN)-3P and ANN-10P models.

Model and dataset	AUROC^a^ (95% CI)	AUPRC^b^ (95% CI)	Accuracy (95% CI)	Sensitivity (95% CI)	Specificity (95% CI)	PPV^c^ (95% CI)	NPV^d^ (95% CI)
ANN-10P							
Training	0.974 (0.968‐0.980)	0.964 (0.956‐0.971)	0.943 (0.935‐0.951)	0.907 (0.890‐0.923)	0.961 (0.954‐0.968)	0.920 (0.903‐0.937)	0.954 (0.946‐0.963)
Validation	0.970 (0.958‐0.982)	0.960 (0.946‐0.973)	0.923 (0.908‐0.937)	0.889 (0.860‐0.918)	0.939 (0.923‐0.956)	0.878 (0.846‐0.911)	0.945 (0.928‐0.962)
Testing	0.968 (0.956‐0.98)	0.936 (0.918‐0.953)	0.927 (0.915‐0.939)	0.873 (0.841‐0.904)	0.947 (0.935‐0.959)	0.853 (0.819‐0.888)	0.954 (0.941‐0.968)
ANN-3P							
Training	0.953 (0.945‐0.960)	0.934 (0.924‐0.943)	0.910 (0.901‐0.919)	0.866 (0.846‐0.885)	0.932 (0.922‐0.942)	0.863 (0.843‐0.883)	0.934 (0.923‐0.943)
Validation	0.954 (0.906‐0.945)	0.938 (0.921‐0.954)	0.908 (0.892‐0.924)	0.892 (0.864‐0.918)	0.916 (0.899‐0.933)	0.841 (0.809‐0.872)	0.945 (0.930‐0.959)
Testing	0.943 (0.927‐0.956)	0.891 (0.869‐0.913)	0.883 (0.867‐0.899)	0.878 (0.845‐0.909)	0.885 (0.866‐0.903)	0.731 (0.691‐0.771)	0.953 (0.940‐0.965)

a AUROC: area under the receiver operating characteristic curve.

b AUPRC: area under the precision-recall curve.

c PPV: positive predictive value.

d NPV: negative predictive value.

## Discussion

### Principal Findings

Epidemiological studies indicate that approximately 10% of the global population faces a lifetime risk of developing kidney stones [1], and approximately 80% of those affected experience recurrence after stone treatment [15]. Although kidney stones are not malignant such as cancers, they can cause severe flank pain that often drives patients to seek emergency care [16]. In more serious cases, stones may lead to urinary tract infections, sepsis, and even hospitalization. Furthermore, recent studies have reported a strong association between nephrolithiasis and the development of chronic kidney disease [4]. Despite these risks, most kidney stones are only diagnosed after patients present with symptoms and undergo imaging-based evaluations in clinical settings [17]. Currently, there is no widely adopted, large-scale, low-cost, and simple screening tool available for the early detection of kidney stones [18,19].

In 2024, our team developed an AI-based model using routine health checkup data to screen for kidney stones in overweight and obese individuals. The model achieved excellent performance (AUC=0.96), and its advantages include low cost, rapid computation, minimal computational power requirements, and no need for physician involvement [6]. This enables its application in frontline health screenings to identify high-risk individuals for further evaluation. Building on the success of our previous work, this study extends the application of our AI framework to the general population. Our findings suggest the potential for a scalable, accessible, and noninvasive tool to detect clinically significant kidney stones across diverse patient groups, providing a much-needed advancement in the proactive management of urolithiasis.

In some regions, certain high-end health screening programs incorporate renal ultrasound (US), plain abdominal radiography (KUB), or even CT to detect asymptomatic kidney stones. While US is a fast, user-friendly, and radiation-free imaging modality, its accuracy is highly operator dependent. Studies comparing US with CT have reported widely varying sensitivity (24%‐69%) and specificity (53%‐90%) levels [20-22]. This variability limits its reliability, especially in large-scale screening, as it requires experienced physicians and access to ultrasound equipment, both of which are resource intensive. Plain x-rays or KUB, although useful in identifying some kidney stones, expose patients to radiation (0.7 mSv per scan) [20]. Plain abdominal x-rays (KUB) can identify some radiopaque stones but have limited sensitivity and expose patients to ionizing radiation (~0.7 mSv per scan) [22] . For women of reproductive age, pregnancy status must be considered before imaging. Furthermore, the interpretation of KUB relies on trained radiologists, making it less practical for community-based screening. Currently, abdominal CT is the gold standard for diagnosing kidney and ureteral stones. However, it has notable drawbacks, including significant radiation exposure (10 mSv for standard noncontrast CT and 1‐3 mSv for low-dose CT) [20,23] and high medical cost [24]. These factors make CT unsuitable for large-scale or repeated screening, particularly in asymptomatic populations. Given the limitations of traditional imaging modalities in mass screening contexts, our AI-based approach offers a compelling alternative. Using only routine clinical parameters from standard health checkups, our model provides a rapid, low-cost, and accessible method for the early detection of kidney stones without the need for imaging, radiation, or specialized personnel. This has significant implications for population-level screening and preventive nephrology.

To the best of our knowledge, this study is the first to use a large-scale dataset that integrates simple clinical information from both patients with and without kidney stones, applying ML techniques to develop a computer-aided screening tool for detecting stones of various compositions. This approach reflects real-world clinical scenarios and aims to support early detection and decision-making for further diagnostic evaluation. In this study, we expanded upon our previous work by incorporating 10 easily accessible clinical variables to construct an AI-based model capable of identifying individuals at high risk for kidney stones [6]. These variables, readily available during routine health checkups, emergency visits, or outpatient consultations, can be collected and analyzed quickly, with no additional burden on patients or health care resources.

One of the key advantages of this tool lies in its simplicity and cost-effectiveness. Unlike imaging-based diagnostics, which require expensive equipment and specialized interpretation, our AI model only needs a standard internet-connected computer and does not rely on urologists or radiologists for execution. This enhances its feasibility and scalability, particularly in resource-limited settings or rural areas. Conceptually, our AI screening model functions similarly to the fecal occult blood test used for colorectal cancer screening [25]—offering a rapid, noninvasive, and low-cost method to identify high-risk individuals who would then proceed to confirmatory imaging tests such as ultrasound, x-rays, or CT scans.

Furthermore, because our model relies on only 10 standard health checkup parameters, patients do not require any additional testing. This makes the tool highly compatible with existing clinical workflows. Given its accessibility and minimal operational requirements, it could be readily implemented in annual health screenings or even in the follow-up of patients who have undergone treatment for kidney stones [26,27]. By enabling earlier detection of stones—even when they are still small and asymptomatic—the model could facilitate timely intervention, reduce the risk of complications, and simplify surgical management.

Importantly, this approach also holds significant potential for use in telemedicine. By leveraging basic clinical data and our AI software, health care providers could remotely screen for kidney stones in patients living far from medical centers, enabling early identification and reducing the likelihood of emergency presentations. Overall, this screening tool may serve as a practical and scalable solution for improving access to care, minimizing disease burden, and promoting proactive management of urolithiasis across diverse health care settings.

Our AI model is designed as a practical screening tool to identify kidney stones using only routine blood and urine data collected during health checkups. In real-world workflows, it can be seamlessly integrated into existing laboratory information systems, allowing automated risk predictions once test results are available. For individuals with a high predicted probability of nephrolithiasis, clinicians can proceed with confirmatory imaging such as ultrasonography or CT for diagnosis, while those with low risk can safely avoid unnecessary imaging. This stepwise approach facilitates early detection and appropriate resource allocation. Furthermore, it should be noted that the algorithm only uses laboratory data from routine urinalysis and no additional (expensive) tests are required. As such, the extra cost for using our algorithm is minimal, making it highly affordable. Early identification enables timely lifestyle interventions or pharmacologic prevention, potentially reducing emergency visits and treatment costs associated with advanced stone disease.

The variables selected in our model were based on previously validated clinical factors strongly associated with nephrolithiasis. Although several of these factors showed significant differences (*P*<.001) between cases and controls, which may contribute to potential overestimation of model performance, we minimized this bias by applying stratified sampling and reweighting techniques during model training. Consistently high AUC and accuracy values across all subgroups further support the robustness and generalizability of our model.

As every participant in this study underwent imaging confirmation to verify the presence or absence of stones, conducting multiethnic and cross-national validation would require substantial resources and logistical coordination. Therefore, this work focuses on an Asian cohort as an initial step. Although our dataset was comprehensive—comprising a large number of patients from multiple hospitals—and we further validated the model using an independent testing cohort in a clinical trial setting, one important limitation must be acknowledged. All patients included in this study were of Asian ethnicity. As a result, the predictive performance of our AI model may be limited when applied to populations of different racial or ethnic backgrounds. Differences in genetics, diet, and environmental factors could potentially affect the clinical presentation and risk profiles of kidney stone disease. To reduce racial and environmental bias, variables known to vary with ethnicity or diet—such as urinary calcium, oxalate, citrate, or lifestyle factors—were excluded. Instead, our model incorporated clinically relevant indicators of stone risk, including hematuria, bacteriuria, and serum creatinine. These features are biologically plausible and less population dependent, suggesting that the model may be applicable across diverse groups, although future international validation is warranted to confirm its performance. Future research should focus on collecting and integrating data from diverse populations to further refine and recalibrate the model. This will be essential to enhance the generalizability and global applicability of the AI-based kidney stone screening tool.

### Conclusions

In this multihospital study, we developed an ML-based model that accurately identifies clinically significant kidney stones using only 10 routine clinical and urine parameters. This low-cost, noninvasive tool enables large-scale, manpower-free screening and can be readily integrated into health checkups or telemedicine, offering a practical solution for early detection and proactive management of nephrolithiasis.

## Supplementary material

10.2196/80764 Multimedia Appendix 1 Details regarding the construction of the artificial neural network model.

## Abbreviations

AI: artificial intelligence
ANN: artificial neural network
AUC: area under the curve
AUPRC: area under the precision-recall curve
AUROC: area under the receiver operating characteristic curve
CT: computed tomography
eGFR: estimated glomerular filtration rate
HPF: high-power field
KUB: kidney, ureter, and bladder
ML: machine learning
RBC: red blood cell
RF: random forest
ROC: receiver operating characteristic curve
SG: specific gravity
US: ultrasound
