# Supplementary material for: Screening for Clinically Significant Nephrolithiasis Based on Simple Health Checkup Clinical and Urine Parameters in General Populations: Multicenter Machine Learning Study
Source: JMIR Med Inform. 2026 Feb 19;14:e80764. doi: 10.2196/80764 (PMC12919908; doi:10.2196/80764)
Supplement: Multimedia Appendix 1 [file medinform-v14-e80764-s001.docx]

**Supplementary Methods**

**Details regarding the construction of the ANN model**

The ANN model was developed in three steps. First, ten models were developed by applying the 10-fold cross-validation protocol on the training data set. More specifically, the training data set was divided into ten subsets, labeled one to ten. At the $i^{th}$iteration, the $i^{th}$ subset was withheld for internal validation while model $M_{i}(x)$ was trained based on the remaining nine subsets. Each model $M_{i}$ had the following form

$M_{i}\left( x \right)=\frac{1}{1+e^{f_{i}\left( x \right)}}$ $i=1,2,\cdots, 10$,

where $x$ represents the input vector of the eight decision variables. The nonlinear function $f_{i}(x)$ was obtained using a multi-layer fully connected neural network. Each layer, except the output layer, consists of three components in concatenation: batch normalization^S2^, affine transformation, and activation, which is illustrated in Figure S1. The output layer had no activation component. This is a standard layer structure of artificial neural structure^S3^. Each $f_{i}(x)$ function was constructed by a network of nine layers including the output layer. In total, each model had 27009 trainable variables. The trainable variables were optimized based on minimizing the binary cross-entropy function. The Adam optimizer^S4^ with recommended parameters was used for the optimization of these variables.

In the second step, the training dataset was further divided into two complementary subsets, one consisting of all male subjects and the other all females. Then, the average of the ten models; that is $M_{av}\left( x \right):=\frac{1}{10}\sum_{i=1}^{10} M_{i}(x)$, was applied to these sets to obtain receiver operating characteristic (ROC) curves for male and female subjects, respectively. Based on the Youden’s indices^S5^ of the ROC curves, separate optimal cut-off thresholds for male and female subjects were determined.

In the last step, two piece-wise affine transformations (the “re-scalers”) were found to unify the separate cut-off thresholds, and to normalize them to the value of 0.5. Denote the thresholds for males and females found in the second step by $T_{m}$ and $T_{f}$, respectively, and the re-scalers for males and females by $S_{m}$ and $S_{f}$, respectively. Then, $S_{m}$ and $S_{f}$ would have the following mathematical expressions

$$S_{m}\left( y_{m} \right)=\frac{0.5}{T_{m}}{\times y}_{m}+max\left\{ 0,\left( y_{m}-T_{m} \right) \right\}\times\left( \frac{0.5}{1-T_{m}}-\frac{0.5}{T_{m}} \right)$$

$$S_{f}\left( y_{f} \right)=\frac{0.5}{T_{f}}\times y_{f}+max\left\{ 0,\left( y_{f}-T_{f} \right) \right\}\times\left( \frac{0.5}{1-T_{f}}-\frac{0.5}{T_{f}} \right)$$

where $y_{m}$ and $y_{f}$ denote the predicted values that model $M_{av}$ produces for a male and a female subject, respectively, and $max\left\{ a,b \right\}$ denotes the larger value between values $a$ and $b$. The concatenation of $M_{av}$ and the re-scalers produces the ANN model $M_{ANN}$, for which the cut-off threshold for classification is calibrated to 0.5 for both male and female subjects.

**Supplementary References**

S1. Levey AS, Stevens LA, Schmid CH, et al. A new equation to estimate glomerular filtration rate. Ann Intern Med 2009; 150(9): 604-12.

S2. Ioffe S, Szegedy C. Batch normalization: Accelerating deep network training by reducing internal covariate shift. Paper presented at: International conference on machine learning 2015.

S3. Goodfellow I, Bengio Y, Courville A. *Deep learning.* MIT press; 2016.

S4. Kingma DP, Ba J. Adam: A method for stochastic optimization. *arXiv preprint arXiv:14126980.* 2014.

S5. Ruopp MD, Perkins NJ, Whitcomb BW, Schisterman EF. Youden Index and optimal cut‐point estimated from observations affected by a lower limit of detection. *Biometrical Journal: Journal of Mathematical Methods in Biosciences.* 2008;**50**(3):419-430.

**Supplementary** **Figure S1**.

Illustration of the structure of a layer.

structure of a layer:

Input

Batch normalization

Affine transformation

Activation input==========

Output
